# The Role of Social-Ecological Resilience in Coastal Zone Management: A Comparative Law Approach to Three Coastal Nations

**DOI:** 10.3389/fevo.2019.00410

**Published:** 2019-10-25

**Authors:** Ahjond Garmestani, Robin K. Craig, Herman Kasper Gilissen, Jan McDonald, Niko Soininen, Willemijn J. van Doorn-Hoekveld, Helena F. M. W. van Rijswick

**Affiliations:** 1 U.S. Environmental Protection Agency, Office of Research and Development, Cincinnati, OH, United States; 2 Utrecht Centre for Water, Oceans and Sustainability Law, Utrecht University School of Law, Utrecht, Netherlands; 3 Wallace Stegner Center for Land Resources, University of Utah S.J. Quinney College of Law, Salt Lake City, UT, United States; 4 Global Change and Sustainability Center, University of Utah, Salt Lake City, UT, United States; 5 School of Law and Centre for Marine Socioecology, University of Tasmania, Hobart, TAS, Australia; 6 Faculty of Law, Helsinki Sustainability Science Institute (HELSUS), University of Helsinki, Helsinki, Finland

**Keywords:** social-ecological resilience, coastal zone management, environmental change, law, environmental governance

## Abstract

Around the globe, coastal communities are increasingly coping with changing environmental conditions as a result of climate change and ocean acidification, including sea level rise, more severe storms, and decreasing natural resources and ecosystem services. A natural adaptation response is to engineer the coast in a perilous and often doomed attempt to preserve the status quo. In the long term, however, most coastal nations will need to transition to approaches based on ecological resilience—that is, to coastal zone management that allows coastal communities to absorb and adapt to change rather than to resist it—and the law will be critical in facilitating this transition. Researchers are increasingly illuminating law’s ability to promote social-ecological resilience to a changing world, but this scholarship—mostly focused on U.S. law—has not yet embraced its potential role in helping to create new international norms for social-ecological resilience. Through its comparison of coastal zone management in Australia, Finland, and the Netherlands, this article demonstrates that a comparative law approach offers a fruitful expansion of law-and-resilience research, both by extending this research to other countries and, more importantly, by allowing scholars to identify critical variables, or variable constellations associated with countries’ decisions to adopt laws designed to promote social-ecological resilience and to identify mechanisms that allow for a smoother transition to this approach. For example, our comparison demonstrates, among other things, that countries can adopt coastal zone management techniques that integrate social-ecological resilience without fully abandoning more traditional engineering approaches to adapt to environmental change and its impacts.

## INTRODUCTION

We face a world where climate change, ocean acidification, species extinction, and changing precipitation patterns are increasingly affecting human well-being. Despite these realities, law plays an important role in promoting human well-being despite these changing realities—that is, of promoting communities’ resilience to environmental change. Coastal communities around the globe are already coping with significant changes from sea level rise, more frequent and increasingly severe coastal storms, and the progressive loss of coastal resources such as coral reefs and fisheries, as warming and acidifying waters interact with pollution and other stressors to severely degrade coastal ecosystems. Coastal zone management (CZM) provides a global focus for research on how law can effectively promote social-ecological resilience to the changes coastal communities are facing.

Over the past several decades, resilience theory and ecological resilience (Holling, 1973) have emerged as powerful tools for understanding the systems through which humans and nature interact, known as social-ecological systems (Berkes and Folke, 2000). Resilience theory describes how dynamic systems operating at a variety of spatial and temporal scales interact with each other, sometimes dampening change, sometimes accelerating it (Walker and Salt, 2006). For example, climate change reflects the fact that greenhouse gas emissions are destabilizing the climate system, a fairly large-scale system both spatially (it operates globally) and temporally (carbon dioxide remains in the atmosphere for centuries). The destabilized large-scale system, in turn, tends to accelerate changes in smaller-scale systems. Thus, warming temperatures both on land and in the ocean prompt species to migrate poleward or to higher elevations, disrupting food webs, and human food security (Craig, 2010).

Within resilience theory, and based on ecological resilience, “social-ecological resilience” refers to the ability of a social-ecological system to absorb change and disturbance without shifting to a new regime with a different set of processes and structures—i.e., without transforming into a new system state (Walker and Salt, 2006). Ecologists have documented repeatedly the ability of systems to transform—for example, prairies shifting from grassland to forest or eutrophication of freshwater lakes. Such transformations, and the threat of more transformations, have critical implications both for human well-being and for resource management (Brown and Williams, 2015).

As a corollary, resilience theory and the documented potential for social-ecological transformations have significant implications for law, governance, and policy (Garmestani and Allen, 2014; Humby, 2014; Benson and Craig, 2017). Law plays an essential role in shaping the discourse regarding social-ecological systems. For example, it helps to frame both how humans perceive their place within these systems and what risks are cognizable and actionable (Garmestani and Allen, 2014; Benson and Craig, 2017). Law can also promote the resilience of desirable social-ecological system states by, for example, mandating reduction of stressors like development and pollution, protecting essential habitat and ecosystem services, or limiting resource extraction to truly sustainable levels (Benson and Craig, 2017).

Over the last decade, research has increasingly focused on the implications of resilience theory for environmental law (Garmestani and Allen, 2014; Humby, 2014; McDonald et al., 2018; Garmestani et al., 2019). Nevertheless, so far, the scholarship exploring this relationship has been fairly limited and nation-centric. For example, previous research has tended to evaluate how well-specific existing laws in particular countries address the underlying features of ecological resilience and to offer recommendations for reducing the tension between social-ecological resilience and law. Moreover, most of this research and scholarship has been based on U.S. law (Ecology and Society Special Issue, 2013; Garmestani and Allen, 2014; Benson and Craig, 2017; Ecology Society Special Issue, 2018; Frohlich et al., 2018; but see McDonald et al., 2018; Wenta et al., 2018), providing little insight regarding the relationship between law and social-ecological resilience more generally. Finally, no scholars to our knowledge have actively engaged in a comparative law approach to assess what the differences and similarities among nations’ legal approaches to similar management issues can teach us about the potential role of law in promoting social-ecological resilience for a changing world.

This article broadens the scope of research about the relationship between social-ecological resilience and the law. It pursues this goal by focusing on a policy issue common to most coastal nations: coastal zone management (CZM) in the face of environmental change. Specifically, this article compares CZM in Australia, Finland, and the Netherlands through the lens of resilience. CZM is a particularly apt subject for such a comparative law exploration because it has a long history of shared approaches to law and policy, facilitated by the widespread participation of coastal nations in the 1982 United Nations Convention on the Law of the Sea and other relevant international commitments such as the U.N. Convention on Biological Diversity, multiple treaties on marine pollution, and shared fisheries management. Advances in the science of ecosystem-based marine management (e.g., United Nations Environment Program (UNEP), 2011) and marine spatial planning (e.g., United Nations Educational, Scientific, and Cultural Organization (UNESCO), 2018) have similarly prompted significant international dialogue and guidance from the United Nations and its various agencies. Moreover, in the face of rising sea levels and increasingly severe natural hazards affecting coasts, many coastal nations are now introducing resilience-based approaches to coastal planning and management (Lloyd et al., 2013; Flood and Schechtman, 2014; Parsons and Thoms, 2017).

Thus, CZM provides a potentially fertile focus area for comparative law studies regarding the role of law in promoting social-ecological resilience: sea-level rise and other aspects of climate change (e.g., worsening or more frequent coastal storms) are already affecting coastal nations around the world; many of these nations engage in CZM and have been doing so for decades; and there are already international norms, best practices, and guidelines for CZM. All these harmonizing developments in the global policy arena suggest that CZM will be a fertile starting point for comparative law research into resilience, because they are likely to reduce the idiosyncrasies between national legal frameworks, thus evading the most pressing challenge for all comparative legal research. We choose in this article to focus on three developed nations that have all engaged in CZM for some time but that have different government structures and that face different risks from climate change. The human population of Australia is concentrated along its coasts and deals with sea-level rise and other risks through a federalist system that divides regulatory authority between the National Government and the individual states and territories. Finland, like Australia, has a long coast, but it is less sparsely populated, with shared responsibilities in CZM between the central government, regional councils and municipalities. The Netherlands is a much smaller and more densely populated country, much of which is already below sea level, resulting in a long-term government focus on preventing inundation, with shared responsibilities between the central government and decentralized governments (provinces, municipalities, and regional water authorities).

As the next sections will explain in more detail, we posit as a normative goal that coastal nations should be seeking to transition to CZM based on an ecological resilience approach—that is, the use of techniques and processes to absorb and adapt to change rather than to resist it. Nevertheless, our assumption at the start of this study was that the three nations we studied would instead all exhibit a strong legal preference for management based on engineering resilience—that is, a reliance on coastal hardening and structures such as sea walls. While that assumption proved accurate in many respects, we also found that all three nations are beginning to experiment with the use of ecological resilience in CZM in response to sea-level rise, potentially reducing coastal adaptation problems several decades or a century from now and suggesting legal mechanisms that other nations could use to progressively transition to an ecological resilience approach. In other words, nations can take advantage of, in particular, sea level rise’s longer time horizon to avoid disruptive and abrupt changes in their CZM laws and policies.

More importantly, this first foray into comparative legal analysis demonstrates the value of such studies in generating a more robust scholarship regarding the role of law in promoting social-ecological resilience to climate change and its impacts. This article therefore ends by suggesting further fruitful avenues of research in this field. For example, comparative analyses like the one we engage in here allow for assessments of whether particular local variables tend to promote engineering or ecological resilience approaches to CZM, as outlined in the next section, or whether the factors that induce a specific nation to adopt a particular CZM approach are idiosyncratic to each country. We hypothesize based on the results of this initial but limited foray into comparative analysis that both general patterns and important individual variations will emerge, and we encourage other researchers to join us in investigating this hypothesis.

## GENERAL LEGAL APPROACHES TO RESILIENCE IN COASTAL ZONE MANAGEMENT: ENGINEERING VS. ECOLOGICAL RESILIENCE

The framing and goals of a country’s CZM policies are critical for how well that nation addresses environmental change. If a nation’s CZM laws seek to protect and preserve the coastal zone in its current configuration and functions, that strategy would reflect an engineering resilience approach (Holling, 1996; Walker and Salt, 2006). Many countries have indeed taken an engineering approach to coastal management, with a focus on increasing the capacity of their coastal zones to *resist* perturbance and change, such as sea level rise and increasing numbers of more severe storms, rather than to *adapt* to such changes. Engineering approaches to CZM tend to result in significant investments in coastal infrastructure, such as dikes, pumps, groins, seawalls, and other coastal armoring (Klein et al., 2001; Baughman and Pontee, 2016; Tetra Tech, 2019).

In contrast, other countries frame their CZM policies to improve the capacity of their coasts to absorb rather than to resist coastal change, reflecting an ecological resilience approach. Ecological resilience, as noted, refers to the capacity of a system to absorb change without transforming into a different state (Walker and Salt, 2006). Accordingly, acknowledging ecological resilience in legal policies necessarily acknowledges the potential for systems to transform. CZM strategies based on ecological resilience assume or acknowledge that the coastal social-ecological systems to which they apply could exist in different states, each with significantly different conditions and providing different ecosystem services (Holling, 1996; Lloyd et al., 2013; Flood and Schechtman, 2014). For example, coastal wetlands and marshes may offer storm protection and promote fisheries by providing extensive nurseries for fish but simultaneously increase the risks of mosquito-borne diseases for coastal populations. On the other hand, filled or “reclaimed” coastal wetlands lower this disease risk and may provide more opportunities for coastal recreation, but simultaneously reduce the capacity of local fish stocks to replenish themselves. Highly productive coral reefs that support tourism and fisheries transform into algae-covered rubble when exposed to warming and acidifying seawater and nutrient pollution.

Governments implementing an ecological resilience approach to CZM generally try to maintain or improve the capacity of coastal social-ecological systems both to adapt to environmental changes and to function at high levels of desirable productivity rather than striving to “freeze” current conditions in place. Such governments might restore and expand coastal wetlands, seagrass beds, mangroves, and other coastal ecosystems both to diffuse the impact of coastal storms and to maintain productive fisheries, or they might enact significant setback requirements and impose rolling easements on coastal properties that require removal of coastal infrastructure as sea levels rise, allowing productive coastal ecosystems to progressively migrate inland. Water law that mandates reductions in the pumping of coastal aquifers can stave off salt water intrusion (Craig, 2018; Delta Program, 2019), while coastal cleanups, land use planning that limits long-term heavy infrastructure in the coastal zones, and improved building codes can reduce the coastal toxic load and hence the public health damage that storms can create (Craig, 2019). Finally, increased numbers of appropriately-located marine reserves can improve the resilience of marine ecosystems, marine biodiversity, and coastal fisheries to changing coastal conditions (Craig, 2012; Delta Program, 2019; Gilissen et al., 2019).

Whether a nation’s CZM strategy is primarily underpinned by engineering resilience approaches or ecological resilience approaches has important ramifications for whether the coastal zones can continue to absorb and adapt to change (Allen et al., 2019). CZM framed (solely) as an engineering and infrastructure (engineering resilience) problem may well-result in the short-term stability of a nation’s coast, but it tends to end with the loss of beaches and associated coastal ecosystems and increased armoring of coastal floodplains (e.g., Kittinger and Ayers, 2010). An engineering approach also risks catastrophic failure of the kind seen in New Orleans during and after Hurricane Katrina. In contrast, an ecological resilience approach to CZM may require coastal communities to migrate, perhaps more than once, in response to changing coastal processes. However, this approach is far more likely to ensure that functional and productive systems continue to exist into the future, even if those systems are transformed. These systems can in turn protect (e.g., storm surge dissipation) and support (e.g., through coastal fisheries) those shifting coastal communities (Kittinger and Ayers, 2010).

As a normative matter, therefore, laws in coastal nations should support the transition to CZM that takes an ecological resilience approach. We emphasize the need for legal *transition* because a nation’s choice of CZM approach raises important trade-offs for law, policy, and politics that will change over time. These trade-offs can most basically be conceptualized as short-term social stability vs. long-term social-ecological productivity. How long the “short term” stable phase can last will often be critical to how governments and governance systems respond to coastal change. Moreover, the transition to ecological resilience approaches can legitimately take longer in some nations without substantially risking damage to either coastal communities or coastal ecosystems as a result of sea-level rise, other climate change impacts, or ocean acidification. Nevertheless, unless a coastal nation is so fortunately situated that it experiences no or very minor impacts from these drivers, eventually engineering approaches will cease to work and may even leave coastal communities worse off than if the infrastructure had never been built. Thus, the legal and management transition will (eventually) become necessary for most coastal countries.

## DISTINGUISHING THE GENERALIZABLE FROM THE IDIOSYNCRATIC IN NATIONS’ CZM LAWS AND POLICIES: THREE CASE STUDIES

Scientific projections regarding coastal social-ecological stability depend on a range of location-specific considerations, including the pace of local sea level rise and ocean acidification and the cumulative and synergistic risks to infrastructure or ecological assets. Such assessments are becoming more common and more accurate as the scientific community grows increasingly skilled at downscaling and localizing global climate change projections. However, given the variations among both social-ecological and cultural realities in the world’s coastal nations, the social dimension of social-ecological resilience is also critical. For example, disaster resilience is one category of approaches to using law to promote social-ecological resilience in CZM. Disaster resilience has found traction in Australia, the United Kingdom, and the United States (Parsons and Thoms, 2017). Disaster resilience assessments rely primarily on *social* variables and conditions to judge a community’s capacity to cope with disasters (Cutter et al., 2008). Nevertheless, disaster resilience approaches typically undervalue ecosystems and ecosystem dynamics, and thus CZM law and policy will need a broader approach to promoting social-ecological resilience (Chuang et al., 2018).

Given the number of variables involved, comparative law studies provide a valuable method for assessing not only whether and how coastal nations incorporate ecological resilience framing and techniques into their CZM, but also what variables emerge as critical to those decisions. Comparative studies allow researchers to question regulatory assumptions and to identify recurring dependencies, key variables, and common correlations. Comparative law studies can thus help to elucidate whether certain constellations of variables make it more likely that a nation will adopt ecological resilience approaches, which in turn can help to prompt both international law promotion of such techniques and wider knowledge sharing. Alternatively, such studies could demonstrate that the decision to pursue an ecological resilience approach depends so intimately on a nation’s idiosyncratic social and cultural circumstances that the ecological resilience approach to CZM is unlikely to become an international or global legal norm and that nation-specific work is necessary.

At the start of this study, we hypothesized that the incorporation of ecological resilience into coastal nations’ CZM is not entirely idiosyncratic. Our case studies support this hypothesis. And our analysis suggests that comparative law studies can increase the overall effectiveness of CZM law and policy in a changing world by paving the way for nations that share key variables to also share knowledge, experience, and techniques regarding ecological resilience approaches to CZM, easing the legal transition to that approach.

## Australia: Emerging Efforts to Fit an Ecological Resilience Approach Into Coastal Infrastructure Protection

**FIGURE 1 ∣:**
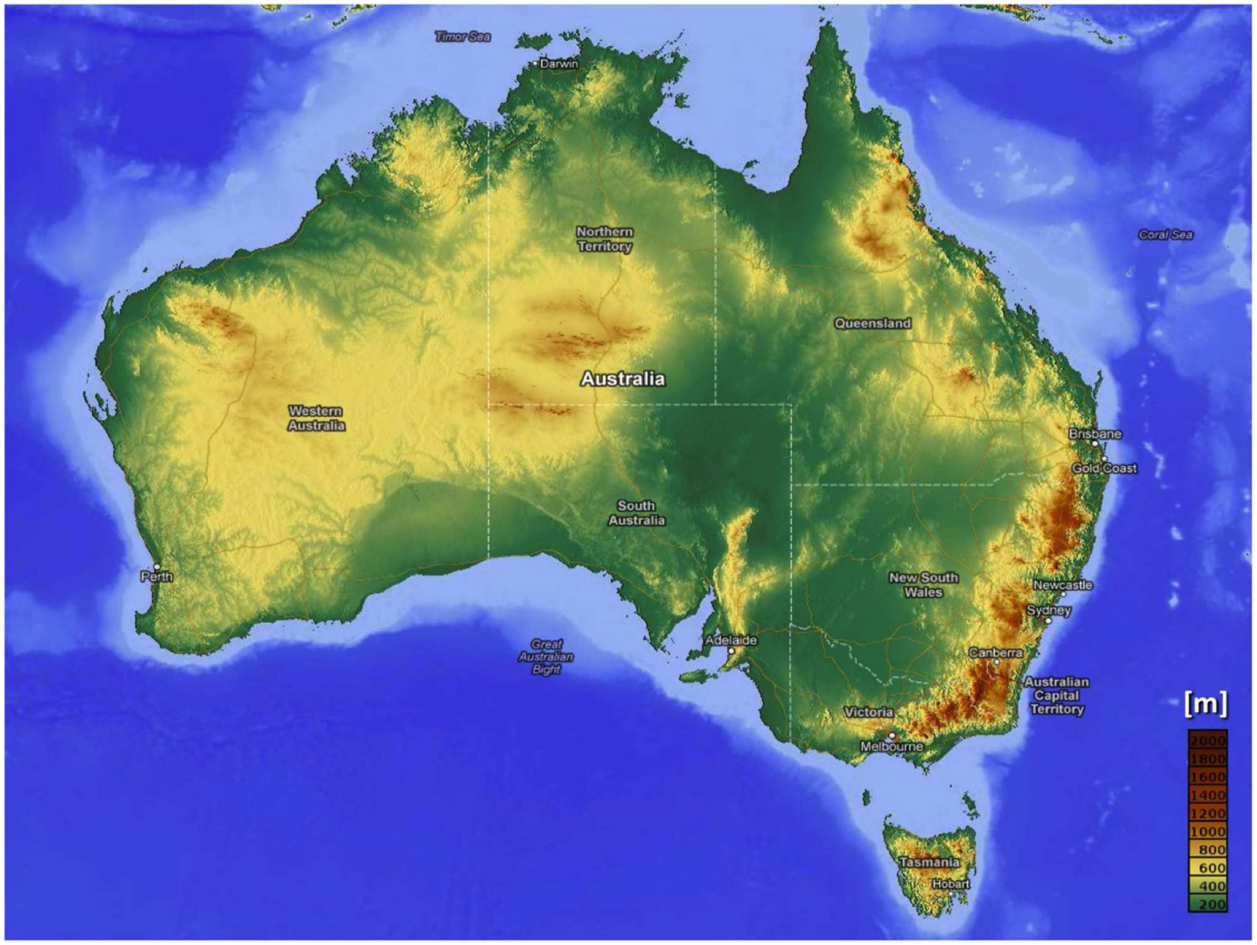
Australia’s topography. The darkest browns are 2,000 m above sea level. Source: https://i.imgur.com/6Ro7UFE.png.

As an island continent, Australia has a vast and varied coastline (Figure 1). Over 85% of the nation’s 24 million people live within 50 km of its 47,000-km coast, particularly concentrated along the east coast in major cities like Sydney, Melbourne, Brisbane and the Gold Coast, and larger regional coastal towns. The coastline is a mix of sandy beaches, rocky cliffs and mangrove or wetlands. Trade-offs between competing coastal uses in populated areas have typically favored intensive development, with associated degradation of coastal ecosystems (Clark and Johnston, 2016). There is extensive existing and high-value infrastructure either on the coastal margins or on low-lying coastal flood plains. This infrastructure includes over 810,000 km of roads, with a replacement value in excess of AUD$60 million. The value of railway lines at risk from coastal climate change impacts is estimated to be AUD$4.9–6.4 billion (DCCEE, 2011). The value of at-risk industrial and commercial infrastructure is over AUD$90 billion and residential infrastructure is over AUD$70 billion (DCCEE, 2011; Kirkpatrick, 2012).

The combination of coastal profiles and population density make Australia particularly vulnerable to the impacts of environmental change (Clark and Johnston, 2016). Beyond the obvious issues of coastal erosion and retreat, increases in the frequency and intensity of estuarine flooding of coastal floodplains is a major challenge (DCC, 2009; Clark and Johnston, 2016). Increased understanding of the likely impacts from sea level rise and extreme events has prompted a re-evaluation of coastal development patterns, at least in greenfield areas. However, the economic and cultural value of this existing legacy development constrains the potential for CZM approaches based on ecological resilience, because powerful political interests promote protection and armoring approaches over retreat.

Under Australia’s federal system of government, the states have the legislative power over coastal management. There is no national CZM strategy or policy, and approaches to CZM are both fragmented and complex. Each coastal state and territory has a combination of laws and policies relating to coastal management, land use planning, conservation, fisheries, and catchment management, which all interact to influence coastal activities. Recent legal reforms have placed ecological resilience and climate change adaptation at the center of coastal management in Australia’s two most populous states—New South Wales and Victoria^1,2^, though the practical implications of these new objectives are yet to be felt.

While there are important legal differences across jurisdictions, the dominant model for dealing with new infrastructure and development involves mapping current and future coastal hazard areas over a range of timeframes and with differing assumptions about projected sea level rise, and then imposing limits on new development in areas identified as being at high risk. In all coastal states except New South Wales, the state government has adopted a sea level rise planning benchmark, which land use planning authorities are required to apply. The level varies across states but is generally set at about a 1.0 m rise above current sea levels by 2,100. New South Wales leaves the determination of what is an appropriate sea level rise planning benchmark to individual municipalities, which has resulted in significant legal variation depending on the property industry’s influence and the local councilors’ acceptance of climate change science within a specific municipality (McDonald, 2015).

States treat existing coastal development differently. Many coastal cities are already protected by seawalls, groins, and regular sand nourishment programs instituted after historical erosion events. A small number of regional coastal municipalities have introduced policies that require the removal of buildings affected by erosion in order to allow for coastal retreat (Foerster et al., 2015). In practice, however, the high value of beachfront properties has created intense political pressure in favor of protecting these exposed properties. At least one coastal authority has reversed its retreat policy, and governments mandate removal of structures only when sudden erosive events actually undercut houses so that they present an imminent threat to public safety (Macintosh et al., 2014; Foerster et al., 2015; McDonald, 2015). More often, media coverage of the homeowners’ plight prompts emergency sandbagging and political promises of long-term protection. Together, these policies and reactions amount to a *de facto* engineering resilience approach. Indeed, in some cases, insurers have even overlooked policy exclusions for coastal hazards and paid out claims resulting from severe storm erosion, further entrenching an expectation that owners can repair or rebuild their properties in the same place.

Current engineering approaches also often squeeze coastal ecosystems. Coastal wetlands and heathlands have already experienced dramatic modification to allow for coastal development (McDonald and Foerster, 2016). Even when a particular location retains a ribbon of vegetation, the relevant laws and governments have made no allowance for natural inland migration in response to changed coastal conditions. In many cases, moreover, such migration would require removal of infrastructure on the landward side of such coastal reserves.

Of course, there are exceptions. For example, governments like the island state of Tasmania have acquired exposed properties when they come to market. This expanding government ownership creates greater flexibility when the time comes to implement a larger retreat strategy. Innovative approaches that align with a social-ecological resilience framing also include spatial planning designations of areas as “future coastal refugia” and limits on what development may occur on such sites (McDonald et al., 2018). So far, however, laws that promote an ecological resilience approach to CZM in Australia remain quite limited.

## Finland: A Focus on Flood Protection and Resilience

**FIGURE 2 ∣:**
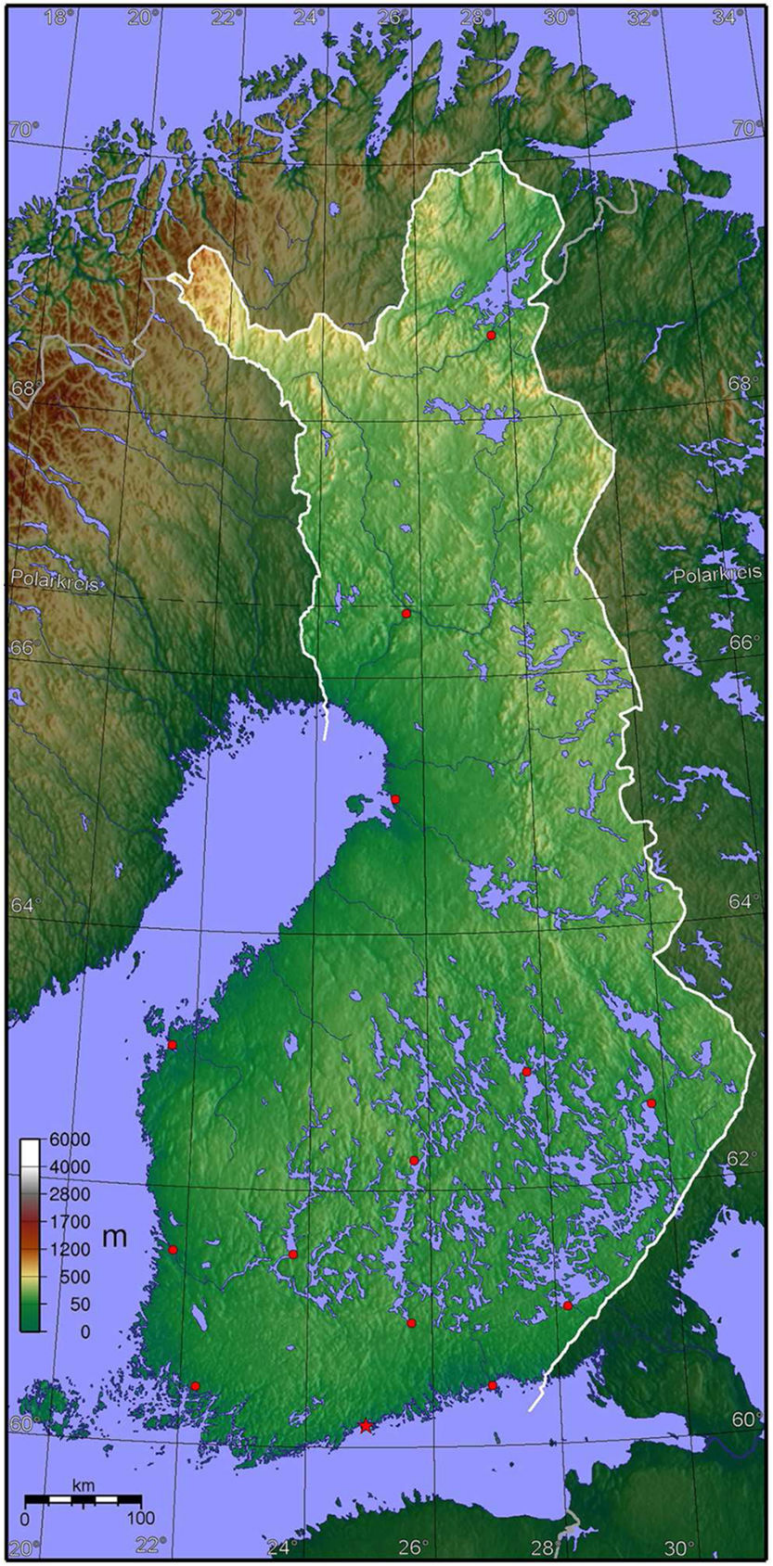
Finland’s topography. Most of the country of Finland rises no more than 50 m above sea level. Source: http://mapsof.net/uploads/static-maps/finland_topo_blank.jpg.

Finland is located on the northeastern bank of the Baltic Sea and has an extensive indented shoreline of 46,000 km (Granö et al., 1999). The shoreline varies considerably, ranging from cliffs and moraine shores to gravel and sandy beaches (Granö et al., 1999). About 32% of the total length of the shoreline is dedicated to housing development and ~1.5% to port and industrial development (Granö et al., 1999). The shoreline also hosts a series of nature conservation sites that are protected under European Union (EU) and domestic nature conservation law (Ministry of the Environment, 2006). Topographically, the coastal areas on the landward side of the UN Law of Sea Convention baseline are flat and prone to flooding (Figure 2). Roughly half of Finland’s 5.5 million people live within 20 km of the sea shore (Ministry of the Environment, 2006).

Environmental change is driving sea level rise in the Baltic Sea. From a coastal management perspective, that sea level rise is partly offset by isostatic land uplift—i.e., the fact that the land is rising (Finnish Meteorological Institute, 2018). This mechanism is a legacy of the last ice age that ended roughly 11,500 years ago, when a thick glacier covered Finland (Finnish Meteorological Institute, 2018). Under the weight of this immense mass of ice, the ground condensed and sank. With the glacier largely gone, the ground is rising again; geologists expect the southern coast of Finland to rise about 40 cm and the northwestern coast about 90 cm over the next 100 years (Ministry of Agriculture and Forestry, 2005; Finnish Meteorological Institute, 2018). This natural mechanism will partly shield the social-ecological systems along the Finnish coasts from the adverse effects of rising sea levels—almost entirely along the northwestern coast but only partially along the southern coast. The capital city of Helsinki, located in southern Finland, concluded that, currently, the land uplift counters sea-level rise almost entirely, but also that sea level rise will become the more dominant phenomenon toward the end of the century (City of Helsinki, 2008).

Despite the shielding effect of land uplift, the Finnish coasts are expected to suffer from increased coastal flooding as a result of rising average temperatures, increased precipitation, snowmelt, and extreme weather events (Ministry of the Environment, 2006) (Figure 2). Especially strong winds combined with meteorological low-pressure areas and coastal currents can cause abrupt and significant sea-level rise and flooding, with harm to infrastructure, utilities, housing, industry and ecosystems (Ministry of Agriculture and Forestry, 2005).

Finland has adopted several national and municipal adaptation and coastal management strategies. These laws and policies seek, among other things, to minimize and adapt to the negative impacts of coastal flooding (e.g., Ministry of Agriculture and Forestry, 2005; Ministry of the Environment, 2006; City of Helsinki, 2008). These strategies emphasize the importance of planning, preparing for and adapting to coastal floods, and integrating adaptation strategies across sectors. The main mechanisms for preparing and adapting to coastal flooding are to: (1) steer housing and industrial development away from flood-prone areas; (2) build new and fortify existing flood defense structures; (3) increase the capacity of municipal sewage systems to handle increased urban run-off; (4) increase the percentage of vegetation zones and decrease the percentage of paved urban areas to improve the soil’s capacity to absorb water; and (5) use existing wetlands for flood management (Ministry of Agriculture and Forestry, 2005; Ministry of the Environment, 2006; City of Helsinki, 2008; Centre for Economic Development Transport the Environment in the Uudenmaa region, 2015). Thus, flood prevention policies in Finland already combine engineering resilience approaches—flood defense structures and sewage systems—with ecological resilience approaches, including development avoidance and the use of soil and wetlands to reduce flooding. Many of the ecological resilience approaches are, however, still at an experimental stage, and need to be upscaled in order to adapt to increasing coastal flooding toward the turn of the century.

Like Australia, CZM implementation in Finland is divided between several state and municipal actors and legal instruments. In flood protection, the two main instruments are land-use planning and flood risk management planning. As elsewhere, land-use planning’s main objective is to steer the geographical location of housing, utilities, and industrial developments into preferred places. Land-use planning in Finland is divided between the state, regional, and municipal actors. These plans range in a hierarchical order from less to more specific: (1) national land-use objectives (national government); (2) regional plans (regional councils); (3) municipal master-plans; and (4) municipal detailed plans (Ministry of the Environment, 1999 p. 132). Regional and municipal plans are especially important in steering new housing and industrial development away from flood-prone areas (Ministry of Agriculture and Forestry, 2005; Ministry of the Environment, 2006).

Flood risk management planning is based on the EU Floods^3^. Such planning is science-based and incorporates: (1) an assessment of the likelihood of floods; (2) societal flood preparedness; and (3) societal recovery after a flood (Finnish Environment Institute, 2013). The main idea in flood management planning is that no new housing and industrial development should be allowed in flood-risk areas. These areas are mapped under the flood management planning regime, the results of which must be considered in planning and permitting new residential and industrial development. Flood management planning integrates the most up-to-date climate and flood models into land-use planning and other government and municipal actions. In the capital area of Helsinki, for instance, most flood-management measures planned and new building permits issued are based on flooding levels that occur, in statistical terms, every 250 years (Centre for Economic Development Transport the Environment in the Uudenmaa region, 2015). Translated into current mean water levels, this safety margin allows new infrastructure to cope with sea level rise of 0.87 m, or 34.25 inches (Centre for Economic Development Transport the Environment in the Uudenmaa region, 2015).

Nature conservation also plays a vital role in Finland’s CZM. Traditionally, all nature conservation strategies relied on a static approach seeking to shield ecosystems from change (Aapala et al., 2017). This strategy is becoming increasingly problematic in light of ongoing environmental change, and new approaches are needed. Current research emphasizes the need for “climate smart conservation,” which evaluates the impact of environmental change on protected species and areas and then adapts protective measures accordingly (Aapala et al., 2017). This approach has yet to become mainstream in either EU/Finnish nature conservation in general or CZM specifically. Nevertheless, the identification of best practices for more ecologically resilient nature conservation policies is currently underway (Aapala et al., 2017).

In sum, Finland’s adaptation strategies and coastal management have relied on the natural land uplift that has until recently compensated for all or most of sea level rise, as well as some of the negative impacts of coastal flooding. As this natural benefit becomes increasingly less effective, however, Finland is developing more active measures that span the engineering and ecological resilience spectrum to deal with environmental change. Engineering resilience is present in the state and municipal strategies and plans to build new and fortify existing coastal flood protection infrastructure, as well as in efforts to increase the capacity of municipal drainage systems to deal with increased precipitation and urban runoff. In addition, current nature conservation policies and laws are based on an engineering approach because they seek to shield protected areas and species from any adverse impacts from climate change.

Ecological resilience approaches are most visible in policies to reduce the percentage of urban paved areas and to promote nature-based solutions, such as using existing wetlands to help manage floods. Steering new development away from flood-prone areas can also be considered an ecological resilience approach because it allows the natural coastal environment to deal with and adapt to sea level rise and coastal flooding. However, this strategy is often not available in developed areas, because existing housing and industrial permits commonly enjoy legal finality and cannot be re-evaluated or modified in light of new scientific knowledge about sea level rise and flood risks. This remains one of the most pressing challenges for shifting existing infrastructure onto a more climate resilient path.

## The Netherlands: Nascent Ecological Resilience Approaches in the Face of an Existential Threat

**FIGURE 3 ∣:**
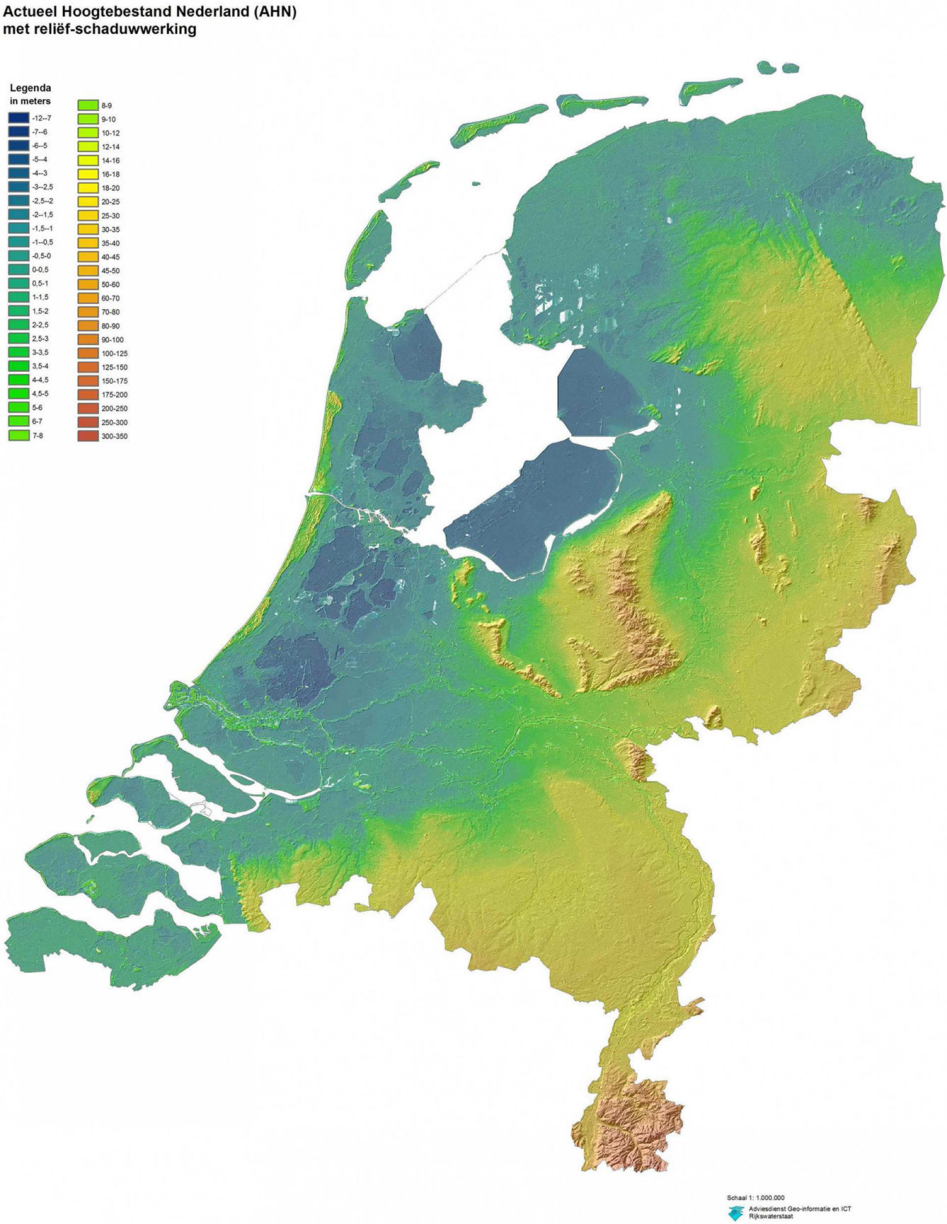
The Netherlands’ topography. The darkest blue areas are 7–12 m below sea level, while the deepest red areas reach 350 m above sea level. Gold areas are 25–40 m above sea level. Source: Netherlands Topographic 3D Map MakerEdChallenge 2 0 by mitrasmit, thingiverse.com.

A delta region located in the northwest of continental Europe, 18% of the Netherlands’ territory (41,526 km^2^) is covered by water (Van de Ven, 2003). Over 35% of the country, including 65% of its population (of a total of 17,358,662) and invested capital (GNP of roughly $740 billion) is currently flood prone, with about one-third of these areas already situated below sea level (Van Rijswick and Havekes, 2012; also see Figure 3). As a result of a centuries-long struggle with water, a highly dedicated and technocratic flood risk governance structure developed in the Netherlands (Kaufmann et al., 2016), testifying to a deeply engrained, and prevailing cultural/political norm to prevent the hinterland from flooding while maximizing socioeconomic development and habitability of the land (Van Rijswick and Havekes, 2012). In practice, this norm requires the nearly constant drainage of over 3,000 polders and the maintenance of nearly 4,000 km of primary flood defense structures, including the coastal flood defense system.

Sea level rise will strain this system, but fundamental changes in law and policy are unlikely in the short term. Recent estimates indicate that sea level will rise 1.8–2.0 mm per year on average, resulting into a total of 25–80 cm by 2,085 (Royal Netherlands Meteorological Institute (KNMI), 2015). In contrast to Finland, moreover, land subsidence—resulting mostly from peat land compaction in the western parts of the country—is making the Netherlands’ sea level rise problem even worse, although regional estimates differ considerably (Royal Netherlands Meteorological Institute (KNMI), 2015). The effects of climate change stress the current system and might eventually force toward more radical strategies such as large-scale relocations, but the Dutch flood defense strategy will continue to ground future flood risk governance in the Netherlands (Gilissen, 2015; Kaufmann et al., 2016; Delta Program, 2019).

The 523-km-long Dutch coastline stretches from the southwestern peninsulas (Scheldt estuary/Rhine-Meuse delta) to the Wadden Islands/Wadden Sea Region in the north and the Ems-Dollard estuary in the northeast (Figure 3). Although this coastline consists mainly of a nearly continuous stretch of sandy beaches and sand dunes, it also incorporates constructed flood defense infrastructure, such as the world-famous Delta Works (mostly in south-western delta but also including the Afsluitdijk) and industrial areas/sea ports, such as Rotterdam, Vlissingen, Den Helder, and Delfzijl. Coastal towns that are home to a flourishing tourism and recreational sector also dots the Dutch coast. The Dutch government has designated large parts of the coastal system as protected areas—Natura 2000 Areas and/or Ecological Main Corridors—under EU and domestic nature conservation law (Backes et al., 2017). Moreover, the Wadden Sea Region in the north of the country is one of the largest protected wetland areas in the world and has been designated as both a Natura 2000 Area and a UNCESCO World Heritage site.

As noted, the Dutch coastline plays an essential role in preventing the hinterland from flooding and, given its sandy nature, forms a particular domain within the Dutch flood risk governance structure (Van Rijswick and Havekes, 2012). Focusing on the sandy parts of the Dutch coastline, both flood defense and environmental protection are key components of Dutch CZM. Moreover, since the early 1990s, so-called “dynamic management” has been a key concept in Dutch CZM (Stronkhorst et al., 2018). The Dutch Technical Advisory Committee for Flood Defense defined “dynamic coastal zone management” as “managing the coast in such a way that natural processes, whether stimulated or not, can take place undisturbed as far as possible, as long as the safety of the inland area is ensured” (De Jong et al., 2014). The main objective of dynamic CZM is to prevent sand dune systems from eroding further and moving inland, thus maintaining a fixed coastline (CPD, 1990; see also https://www.rijkswaterstaat.nl/kaarten/kustlijnkaart.aspx). Under Article 2.7 of the Dutch Water Act of 2009, Rijkswaterstaat, the Dutch Central Government’s Water Management Agency, achieves this stabilization where possible through flexible mechanisms to foster continued ecological integrity, including coastal ecosystem preservation, the maintenance of specific functions, and species protection based on EU/domestic nature conservation law (De Jong et al., 2014). Rijkswaterstaat’s most common coastal management techniques is near-shore sand nourishment, a so-called “soft engineering” approach, also dubbed a “Building with Nature” approach (Van Slobbe et al., 2013; De Vriend et al., 2015). Through this technique, large amounts of sand are pumped or transported to the shallow waters adjacent to the coast, allowing natural processes (mainly tides, waves, and wind) to gradually transport the sand landward, where it can elevate beaches, stabilize dunes, and, where needed, restore eroded sites and reverse related ecological degradation (Arens and Wiersma, 1994; De Ruig and Hillen, 1997; Van Dalfsen and Aarninkhof, 2009; Stive et al., 2013; De Jong et al., 2014). Dynamic CZM thus strategically aims to create a robust and resilient coastline and dune system that has the capacity to recover from erosion and related damage after storms and storm surges. Thus, the Netherlands pursues its overall engineering resilience goal through a generally effective and efficient strategy that blends engineering and ecological resilience approaches by spurring natural sand replenishment.

Nevertheless, overall, the Netherlands strives to keep its coastline and dune system stable and resistant to natural evolution, an inherently engineering resilience approach to CZM. Indeed, many policy documents use the term “veerkracht” (resilience) to refer to the coast’s ability to bounce back to the status quo. In addition, this engineering resilience approach will not be ending any time soon: with a predicted sea level rise of 0.25–0.80 m by 2085 (Royal Netherlands Meteorological Institute (KNMI), 2015), the Dutch long-term (2,100) adaptation plan calls for intensified sand supplementation (Van Rijswick and Havekes, 2012; Delta Program, 2019), and the first pilot projects have already started (e.g., Project “Sand Motor”; De Schipper et al., 2016).

Moreover, hard engineering approaches remain important backstops to sand supplementation as sand supplementation does not always work to provide the legally required level of flood protection at some locations. These are the so-called “weak links” in the Dutch coastal defense system. At these locations, the relevant regional water management authorities have implemented additional or alternative measures to meet the legal security standards for “primary flood defense structures” (Article 2.4 of the Dutch Water Act 2009), such as building concrete constructions in dunes (Gilissen et al., 2010). In other words, where naturally driven processes fall short of meeting Dutch CZM goals, hard engineering remains a reliable solution, at least in the short to medium term.

Apart from flood protection, dynamic CZM through sand supplementation and other supportive measures (e.g., opening the Haringvliet sluices and flooding the Hedwigepolder) can be beneficial for environmental protection, contributing to the Dutch coast’s and hinterland’s ecological potential. Seven habitat types are present along the Dutch coast, and each is home to many protected and common species (http://natura2000.eea.europa.eu/). As noted, most of these areas are protected under EU and Dutch nature conservation law, which means that the Dutch government must preserve or improve their ecological values (Backes et al., 2017). However, Dutch (and EU) ecological policies have tended to emphasize ecological preservation and focus on saving specific species and ecological statuses, leaving little room for these systems to expand or transform. Thus, even though the Netherlands uses processes such as sand distribution to promote ecological function in large parts of its ecologically relevant coastal zones, the applied strategies still primarily embody an engineering resilience approach.

## Comparative Analysis and Conclusions

Australia, Finland, and the Netherlands are developed nations, and they all have significant financial and infrastructure investments in their coastal zones. In addition, each nation has already significantly altered large swaths of its coastal ecosystems, losing considerable ecosystem function to development. As might be expected, the legal and policy framework of each country favors an engineering resilience approach to CZM that prioritizes the preservation of expensive and important coastal infrastructure, although each nation has also grafted on ecological preservation considerations pursuant to state (Australia), national, and EU (Finland and the Netherlands) law.

As such, the most important finding of this preliminary study is that, despite deep and pervasive historical legal and policy commitments to an engineering resilience approach to CZM, Australia, Finland, and the Netherlands each show signs of an emerging ecological resilience perspective. In Australia and Finland, both countries that still have relatively large amounts of space, this emergence primarily has taken the initial form of steering *new* development away from the coast, reducing future hardening of the coastal zone. The Netherlands, lacking this spatial luxury, has in some senses been far more creative in blending its engineering and ecological resilience perspectives.

Australian settlement consists of concentrated coastal development in urban areas. Law and policy in smaller coastal urban areas purport to favor a coastal retreat strategy, but in practice to date the overall emphasis continues to be on protecting and armoring shoreline infrastructure. This political reality constrains Australian CZM into an engineering resilience approach, at least in its highly urbanized areas. Property owners expect that they will be able to rebuild in the coastal zone after erosion or storm damage, which reflects an engineering resilience norm that seeks to have coastal communities bounce back to how they were before a disaster. With sea level rise and projections of more intense storm events, however, Australia will inevitably have to alter its approach to CZM, and some signs of this needed shift in CZM approach are appearing in New South Wales and Victorian state legislation and the approaches of smaller municipalities. Thus, at least some coastal managers in Australia appear to be adopting a perspective that acknowledges the dynamic nature of social-ecological systems, a nascent ecological resilience approach to CZM.

In Finland, CZM focuses on land use and flood risk planning that also has its roots in an engineering resilience approach. Government officials generally cannot re-evaluate existing development in coastal zones in light of new information (legal finality). Thus, current CZM in Finland leaves little room for adaptation to rising sea levels and flooding in developed areas; as a result, CZM instead must rely on coastal armoring to protect existing structures. Even so, as in Australia, there are signs that social-ecological resilience is seeping into Finland’s CZM. Laws restrict new development in coastal zones, resulting in most new development occurring inland and freeing undeveloped coastal areas to adapt to changing conditions. Finland is also experimenting with nature-based solutions, such as using existing wetlands for flood protection, and with increasing the amount of unpaved coastal urban areas, again strengthening the ability of coastal areas to adapt to changing conditions, such as increased flood risk.

The Netherlands literally has the least space of the three nations studies to absorb change and to adapt to changing conditions, as well as the strongest absolute social need to preserve coastal stability. Because ~65% of the country’s population already resides in flood prone areas, with a significant percentage of the country already below sea level, Dutch CZM is, unsurprisingly, characterized by engineered flood defenses of dikes and canals combined with large protected coastal areas designed to “freeze” the coastal system in a static state. This quintessentially engineering resilience approach to dealing with coastal system dynamics has been baked into Dutch culture and law for centuries.

Even in the Netherlands, however, ecological resilience approaches are emerging, albeit always subordinate to the overarching goal of coastal stability, an approach that some researchers have dubbed “Building with Nature” (Van Slobbe et al., 2013; De Vriend et al., 2015). Natural features such as sand dunes and beaches are legally recognized components of flood protection, meaning that Dutch CZM law and policy recognize the important ecosystem services that these features provide. Moreover, protecting and building up beaches and sand dunes is critical to the nation’s overall CZM strategy. The “soft engineering” technique of sand distribution uses natural processes to ensure that these coastal features and their associated ecosystems and ecosystem services remain intact and well-functioning. In addition, recently there have been efforts to better protect and construct wetlands in order to supplement the system of dikes and pumps that keeps the country dry, hinting that the ecological resilience approach to flood protection is expanding in the Netherlands.

Beyond their individual trajectories, these three nations’ approaches to CZM also suggest that the initial binary that this article proposed, contrasting an engineering resilience approach and an ecological resilience approach to CZM, in fact represents less of a dichotomy for coastal law and policy than a malleable ensemble of tools and strategies. In other words, the two approaches to CZM are not (entirely) mutually exclusive, and legal evolution can allow for the progressive emergence of an ecological resilience approach (see also Cheong et al., 2013). That full-scale legal revolution might not be necessary before a nation can implement the more adaptive approaches to CZM that are increasingly necessary in a changing world is an important finding in and of itself for legislatures and other policymakers (Garmestani et al., 2019).

However, the analysis of these three countries also suggests that legal and policy options for CZM will always be constrained by the physical realities of a particular coastal nation. The fact that sea-level rise is not a significant concern for large stretches of Finland’s coast effectively gives Finland far more flexibility in its CZM approach than either Australia or the Netherlands will be able to tolerate. Essentially, climate change imposes less pressure on Finland to evolve its laws to an ecological resilience approach than it imposes on the Netherlands or, at the end of this extreme, disappearing Pacific island nations, simply because Finland’s land mass is still responding to the retreat of ice-age glaciers.

## IMPLICATIONS FOR FUTURE RESEARCH ON LAW AND SOCIAL-ECOLOGICAL RESILIENCE ALONG THE COAST

As we stated at the beginning of this article, the goal of this research project was not just to compare CZM approaches in Australia, Finland, and the Netherlands but, more importantly, to demonstrate the value of comparative law research in the study of law’s role in promoting social-ecological resilience to changing environmental conditions. As limited in scope as this study is, our comparative analysis of these three countries already suggests several fruitful focal points for future research. For example, the realization that all three countries—admittedly, to different degrees—already deploy ecological resilience strategies and techniques within an overall CZM legal framework that privileges engineering resilience raises several important questions regarding the extent to which nations can and do blend these two approaches and whether blending evolves eventually into an ecological resilience-based approach to CZM. Research assembling a variety of case studies and documenting exactly how coastal law and policy are evolving in a variety of nations could thus provide important contributions to global CZM in the Anthropocene.

The realization that physical realities remain important factors in shaping a particular nation’s CZM law and policy also suggests productive avenues for interdisciplinary research. Specifically, our initial three case studies suggest that the disciplines of legal geography and historical geography have important roles to play in investigating the intersection of resilience theory and CZM and in formulating effective future CZM law for individual nations.

More generally, a proposition to be tested in future research is whether coastal nations typically begin with an engineering resilience approach to CZM (and, indeed, to their environmental laws more broadly). Our three case studies are insufficient to discern, for example, whether this approach is globally typical, or is found mostly in European-derived government systems, or is found mostly in developed nations, or even is idiosyncratic to the three countries we happened to study (plus the United States). We also have not focused on whether the hard engineering approaches pre-dated CZM law and policy (i.e., law and policy reflect a reality that already existed) or occurred outside the law (i.e., CZM *practice* contradicts CZM *law*). If it turns out that countries with significantly different legal traditions and histories (e.g., minimal influence from European colonialism), or with significantly different economic statuses, than the three countries studied here typically employ an ecological resilience approach to CZM, further questions for research would emerge, such as: what factors prompt a coastal nation to adopt an ecological resilience approach to CZM from the beginning? How influential are factors such as a lack of intense coastal settlement and development, the cultural/religious importance of coastal ecosystems, or deeply engrained social norms against building permanent infrastructure along the coast? Can these factors be generalized, or is each coastal nation in important senses unique?

A final consideration worthy of more comparative investigation is the fact that legal systems have different capacities to innovate within their CZM strategies based on factors such as enforcement mechanisms, flexibility in law, the rate of statutory change, and the role of litigation. For example, some legal systems already embrace doctrines that can be harnessed to promote the adaptation and evolution of CZM. In common-law systems derived from England and British colonialism (including the United States, Canada, Australia, New Zealand, and South Africa, plus extensive influence on various African and South American nations), concepts of public and private nuisance, trespass, negligence and strict liability, and in some, public trust, provide mechanisms for evolving natural resources law and policy (Rechtschaffen and Antolini, 2007). As one example, the States of Oregon, California, and Hawai’i in the United States have used the public trust doctrine to require holistic protection of aquatic ecosystems (Craig, 2010; Boisjolie et al., 2017). How do these different capacities affect the CZM approaches that nations take, or the evolution of those approaches in the face of environmental change?

This example also highlights the potential importance of subnational governance, a factor present to some degree in all three countries studies here. Those local, regional and state levels often have greater capacity to innovate because they can provide greater capacity for stakeholder engagement and an appropriate scale for experimental management approaches (Charnley et al., 2018). Nevertheless, super-national law can also be important in spurring innovation. Thus, as two examples, parties to the United Nations Convention on Biological Diversity and EU member nations are subject to ecological obligations that are supposed to influence, and in some cases can supersede, national proclivities toward a purely engineering resilience CZM approach, as is evident in both the Finland and Netherlands case studies here. Future research might well-investigate how governance pluralism, as is prominent in the United States, and hierarchical governance influence the ability of CZM law and policy to adapt to a changing world.

Clearly, different legal framings of resilience in the coastal zone have important implications for future coastal social-ecological resilience in the face of accelerating environmental change (Clarvis et al., 2013). The limited comparison presented here suggests that much fruitful work remains to be done through comparative law approaches to CZM. Specifically, our initial foray into this kind of approach strongly suggests that more extensive interdisciplinary and comparative research could provide coastal nations with numerous policy tools and legal mechanisms for transitioning to ecological resilience techniques for and approaches to CZM that will better promote continued (if transformed) productivity and social-ecological resilience in the face of sea-level rise, worsening coastal storms, warming seas, and ocean acidification.

## Footnotes

1 State of New South Wales, *Coastal Management Act 2016*.

2 State of Victoria, *Coastal and Marine Act 2018*.

3 *Directive 2007/60/EC on the Assessment and Management of Flood Risks*. OJ L 288, 6.11.2007, 27–34.
